# Community contextual predictors of endoscopic colorectal cancer screening in the USA: spatial multilevel regression analysis

**DOI:** 10.1186/1476-072X-9-44

**Published:** 2010-09-03

**Authors:** Lee R Mobley, Tzy-Mey Kuo, Matthew Urato, Sujha Subramanian

**Affiliations:** 1 RTI International, Discovery and Analytical Sciences Division, 3040 Cornwallis Road, PO Box 12194, Research Triangle Park, NC 27709-2194, USA; 2 RTI International, Social and Statistical Sciences Division, 3040 Cornwallis Road, Research Triangle Park, NC 27709-2194, USA; 3 RTI International, Social and Statistical Sciences Division, 1440 Main Street, Suite 310, Waltham, MA 02451-1623, USA

## Abstract

**Background:**

Colorectal cancer (CRC) is the second leading cause of cancer death in the United States, and endoscopic screening can both detect and prevent cancer, but utilization is suboptimal and varies across geographic regions. We use multilevel regression to examine the various predictors of individuals' decisions to utilize endoscopic CRC screening. Study subjects are a 100% population cohort of Medicare beneficiaries identified in 2001 and followed through 2005. The outcome variable is a binary indicator of any sigmoidoscopy or colonoscopy use over this period. We analyze each state separately and map the findings for all states together to reveal patterns in the observed heterogeneity across states.

**Results:**

We estimate a fully adjusted model for each state, based on a comprehensive socio-ecological model. We focus the discussion on the independent contributions of each of three community contextual variables that are amenable to policy intervention. Prevalence of Medicare managed care in one's neighborhood was associated with lower probability of screening in 12 states and higher probability in 19 states. Prevalence of poor English language ability among elders in one's neighborhood was associated with lower probability of screening in 15 states and higher probability in 6 states. Prevalence of poverty in one's neighborhood was associated with lower probability of screening in 36 states and higher probability in 5 states.

**Conclusions:**

There are considerable differences across states in the socio-ecological context of CRC screening by endoscopy, suggesting that the current decentralized configuration of state-specific comprehensive cancer control programs is well suited to respond to the observed heterogeneity. We find that interventions to mediate language barriers are more critically needed in some states than in others. Medicare managed care penetration, hypothesized to affect information about and diffusion of new endoscopic technologies, has a positive association in only a minority of states. This suggests that managed care plans' promotion of this cost-increasing technology has been rather limited. Area poverty has a negative impact in the vast majority of states, but is positive in five states, suggesting there are some effective cancer control policies in place targeting the poor with supplemental resources promoting CRC screening.

## Background

Colorectal cancer (CRC) is the third most common cancer in both men and women, accounting for 10% of all new cancers and 9% of cancer deaths for each [1]. CRC can be detected through endoscopic screening, and survival rates are 90% if diagnosed early. Endoscopic screening can also prevent CRC by detecting and removing precancerous lesions as part of the screening procedure. However, CRC screening rates remain low, as only 42.2% of the over-50 population received any type of CRC screening within the past 5 years [2]. Only 39% of CRC cases are diagnosed at an early stage, and CRC remains the second leading cause of cancer death in the United States [3-6]. CRC incidence is 15 times greater among persons aged 65+ than among younger populations [7]. This is important because the population cohort size and life expectancy of older persons continues to increase [8,9]. Thus, the comorbidity and mortality burdens of CRC in the older population are expected to increase unless enhanced understanding of factors associated with screening uptake can be used to effectively promote screening and early-stage cancer diagnosis.

Endoscopic CRC screening and diagnostic follow-up are cost-effective strategies in the prevention of CRC [10-12]. This means that society values the benefits of CRC screening in terms of reduced morbidity and mortality burdens and is willing to pay the price of screening. Sigmoidoscopy, which observes only the lower portion of the colon, may be more cost-effective than colonoscopy because of large differences in cost. Both procedures are quite effective at detecting cancer or detecting and removing precancerous lesions, thus preventing CRC from progressing. However, colonoscopy is more clinically effective at detecting precancerous lesions, because it observes the entire (upper and lower) colon. As costs of chemotherapy rise with the adoption of new drugs [13] or as guidelines for repeat colonoscopy are updated to recommend longer periods between exams [10], colonoscopy becomes even more cost-effective, even cost-saving over the patient's lifetime.

The cost-effectiveness of endoscopy for CRC screening has resulted in coverage for the elderly under traditional Medicare insurance. Medicare fee-for-service (FFS) is the traditional Medicare insurance available for persons aged 65+ who have earned enough work credits to qualify for Social Security benefits. Part A coverage for hospitalization is available for all as a Social Security benefit, but Part B coverage (for outpatient care) requires payment of a premium (about $360 per month). Traditional Medicare FFS coverage is usually defined to include both Parts A and B coverage; we use this definition in this paper. Traditional Medicare FFS coverage has been consistent with CRC screening guidelines. Since 1998, Medicare FFS has covered sigmoidoscopy every 4 years for all persons over age 50 and colonoscopy every 2 years for persons at high risk for CRC. In 2001, most guidelines suggested that people over age 50 should receive an annual fecal occult blood test (FOBT) or fecal immunochemical test (FIT), periodic sigmoidoscopy, or a combination of FOBT/FIT and sigmoidoscopy. Many organizations also recommended screening average-risk persons with colonoscopy every 10 years. With benefits expansion in 2001, FFS Medicare now covers colonoscopy every 10 years for persons of average risk; however, there is considerable geographic variation in screening uptake [14,15].

Medicare can expect to cover most older individuals for their remaining life spans and can thus recoup the financial benefits from covering the service. By contrast, the financial benefits from promoting a cost-effective screening technology may not accrue to Medicare managed care insurance plans if enrollees do not remain enrolled for a long period of time in the plan. This may reduce the likelihood that the managed care plan will promote use of the service or recommend use concordant with established guidelines [16].

Elderly persons have the option of either enrolling in traditional Medicare or in one of several managed care plans provided by private insurance companies, known as Medicare managed care (MMC) plans. Nationally representative evidence suggests that MMC enrollees were more likely to use the much less costly FOBT procedure than endoscopic procedures for CRC screening, as compared to FFS Medicare enrollees [17]. FOBT can detect cancers that are already established in the bowel and is much less expensive than the more clinically effective endoscopic procedures, which have the added benefit of identifying and removing precancerous lesions. Current guidelines recommend using a combination of FOBT or the newer FIT [18] and endoscopy [19]. We focus on endoscopy use in this paper because Medicare claims accurately capture these services but do not accurately capture all FOBT/FIT test use, and because recent evidence suggests that FOBT/FIT tests are rarely used properly [20].

### Managed Care Spillover Effects

Endoscopic CRC screening is a cost-increasing technology, relative to use of FOBT/FIT alone. When managed care penetrates the health care market, it may impact diffusion of new cost-increasing technologies and the practice patterns of providers in the area, a phenomenon known as *managed care spillover effects*. These effects have been shown to spill over onto constituents who are not enrolled in the managed care plans, such as traditional Medicare enrollees [21]. Our study subjects are traditional Medicare enrollees, and we expect there may be spillovers on their behaviors as follows. Changes in practice patterns can spill over to people who are not insured by the managed care plans, but who are seen by the physicians who are affected by the information or guidelines the plans disseminate. Also, traditional Medicare insured people may compare treatment options and be influenced by the care patterns received by their peers who are in MMC plans. When peers are in managed care plans, their available treatment options may influence their neighbors who are not in managed care plans. Thus, elderly persons with traditional Medicare, living in neighborhoods where many peers are enrolled in MMC plans, may be influenced by the managed care plan enrollees, causing a behavioral spillover. Thus, if MMC plans favor use of FOBT over endoscopy, we might expect to see negative spillovers from MMC penetration on FFS beneficiary use of endoscopy. Two recent studies of endoscopic CRC screening by large samples of Medicare FFS beneficiaries in different time periods find contradictory evidence regarding MMC spillover effects [22,23]. Koroukian et al. [23] concluded that there could be modest MMC spillovers that improve use of endoscopic CRC screening, based on 1999 data covering the traditional Medicare population in the largest US counties. Mobley et al. [22] found that spillovers were positive in some states and negative in others, using 2000-2005 data for 11 states and a 5% sample of Medicare FFS enrollees.

**Figure 1:**
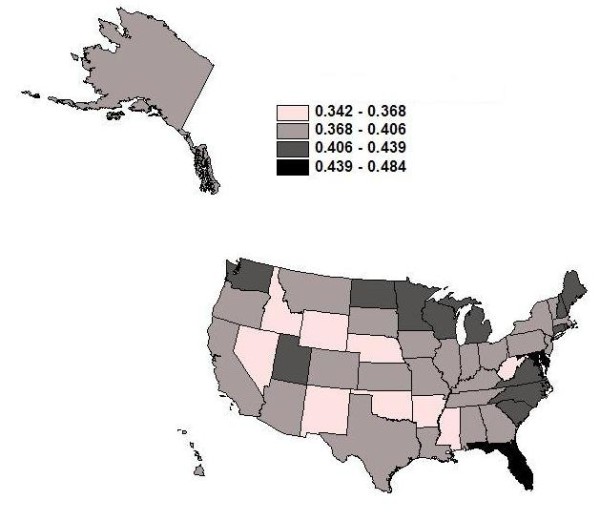
**Utilization Rates for Endoscopy**. Proportion of the 100% Medicare fee-for-service (FFS) population cohort, defined in 2001, who ever utilized endoscopic screening for colorectal cancer during 2001-2005.

The literature suggests that utilization of CRC screening varies widely across geographic areas [14,24-33]. No study to date has examined the entire FFS Medicare population over a period following the 2001 Medicare coverage expansion to include colonoscopy (in addition to sigmoidoscopy), which is a major contribution of this paper. We find considerable variation in our 100% FFS Medicare population screening rates among the states, as shown in Figure 1. The proportions of the population with any use in the 5-year period range from 44+% in Maryland, Minnesota, Delaware and Florida to less than 37% in nine states, with the lowest rate of 34% in New Mexico.

Rates of provider reimbursement for endoscopic procedures vary considerably across insurance types and locations [25-28]. With no consensus regarding which screening protocol is best, local practice patterns may evolve that reflect local insurance reimbursement rates, local physician specialties or capital investments, and managed care practices that spill over onto other sectors, leading to geographic disparities in the type and rate of CRC test utilization [14,22]. Given these market influences, several recent studies have highlighted the need to assess the capacity available to perform endoscopic surveillance to detect CRC [26,29-35]. Because managed care penetration and endoscopic capacity varies so much across the country, and managed care spillovers can impact the diffusion of new cost-increasing technology (endoscopy versus FOBT/FIT), geographic differences in availability of endoscopic services are likely to persist.

## Results

**Figure 2:**
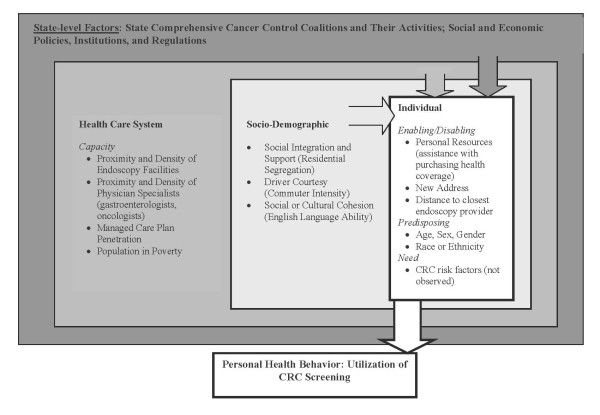
**Socio-Ecological Model**. Socio-ecological model of factors impacting probability of endoscopic colorectal cancer screening.

Our modeling is based on a socio-ecological model describing a comprehensive set of predictors of CRC screening at multiple levels, including personal, social community, health system, and state (Figure 2). The conceptual model situates the individual decision maker into an ecological context that has personal, socio-demographic, and health system factors that interact spatially to influence health utilization behavior.

We define the health system factors at the county level, and these include capacity factors (availability of endoscopy facilities, gastroenterologists, and oncologists) and market factors impacting diffusion dynamics (e.g., MMC penetration, area poverty). The socio-demographic factors are defined for smaller areas known as primary care service areas (PCSA), which were formed from the aggregation of ZIP code tabulation areas to reflect Medicare patient travel to primary care providers [36]. PCSAs are smaller than counties and are thought to better represent local neighborhood conditions [37]. Spatial interaction along the pathways to health care utilization is impacted by socio-demographic factors, including English language ability among the elderly or residential segregation by race or ethnicity; and transportation factors, such as commuter intensity in the local PCSA. Other personal enabling or disabling factors related to transportation impedance are distance to the closest endoscopy provider and whether the person has recently moved to a new ZIP code of residence (see Table 1, which describes the variables and their original sources; many are now available online at the public use RTI Spatial Impact Factor Database Web site: http://rtispatialdata.rti.org).

**Table 1:** Variables Used in Multilevel Regression Analysis

Characteristic of Sample Population	Data Source
*Enabling/Disabling*	Developed from CMS Medicare 100% Denominator Files, 2001-2005, and calculations performed using geocoded data and distance algorithms
Moved to a new ZIP code in same state, 2001-2005	
Months with extra assistance from state Medicaid to purchase Part B insurance, 2001-2005	
Distance (miles) to closest endoscopy facility	
*Predisposing*	
Age in 2001	
Gender; race or ethnicity	
	
**Socio-Demographic Factors (PCSA area)**	
*Social Integration and Support*: Segregation (isolation) index describing residential segregation by race or ethnicity, following Massey and Denton [52]	Developed from US Census 2000 data at ZCTA levels aggregated to PCSAs using ZCTA-PCSA crosswalk: http://rtispatialdata.rti.org
*Stressor, Driver Courtesy*: Commuter intensity reflecting the proportion of the workforce commuting 60 minutes or more each way to work	
*Social or Cultural Cohesion*: Proportion of the population aged 65+ with little or no English language ability, 2000	
	
**Health System Factors (County area)**	
*Capacity*: Average number of endoscopy facilities per thousand population aged 65+ in each person's residential area, defined in 2001	Providers identified from CMS Medicare outpatient claims files; annual census population
*Capacity*: Number of oncologists or per thousand population aged 65+, defined in 2001	Area Resource File and annual census population
*Market factor*: MMC plan penetration, defined in 1998	CMS Geographic Service Area File
*Market stressors*: Proportion of population living below the federal poverty level in 2001; proportion of the county that is rural	Census annual poverty and population

Note: MMC = Medicare managed care; ZCTA = ZIP Code Tabulation Area; HRSA = Health Resources and Services Administration; PCSA = Primary Care Service Area; CMS = Centers for Medicare & Medicaid Services.

Using this conceptualization, we estimate a binary probit model of individual screening behavior separately for each state's Medicare FFS population. Because endoscopic screening is not recommended annually, we define a cohort in 2001 and follow them through 2005 and determine utilization over this 5-year interval. Holding constant statistically the many factors associated with CRC screening utilization, we isolate the independent effects of three contextual variables amenable to policy intervention: MMC spillovers, elderly English language ability, and poverty in the individual's residential neighborhood. We answer the following research questions:

1. Are there significant MMC spillover effects? How do these vary across states?

2. Are there significant elderly language ability effects? How do these vary across states?

3. Are there significant area poverty effects? How do these vary across states?

MMC penetration in one's residential neighborhood and English language ability among the elderly are thought to impact the information flows among seniors. Higher MMC penetration is expected to be associated with larger managed care spillover effects, which could be either positive (increasing screening utilization) or negative, depending on whether managed care spillovers slow the diffusion of the new cost-increasing technology or promote it. Living in a community with poorer English language ability among the elderly is expected to lower the probability of utilization, unless the foreign cultural community has undertaken promotional activities to promote CRC screening. We also focus on a third variable, area poverty, which is expected to affect both the supply of and demand for CRC screening. We anticipate that seniors living in more impoverished neighborhoods will have lower probability of utilization. Even though our study population is insured by traditional Medicare for endoscopic procedures, there are out-of-pocket costs (about $25 for sigmoidoscopy and about $250 for colonoscopy) that may be substantial for poor or financially insecure elderly persons. For clarification, we control at the personal level for elderly who meet the low-income threshold and qualify to receive subsidies from state Medicaid programs to cover their Part B premiums (but subsidies do not cover procedure co-payments). These low-income elderly are dually eligible for Medicare and Medicaid, as they receive benefits from both programs. Holding this dual eligibility indicator variable constant statistically, we still expect to find a significant neighborhood poverty effect. Elderly living in poor neighborhoods are expected to have worse access to endoscopy providers and to be less financially secure and less willing to pay the required co-payments for endoscopy services.

The results for these three policy variables are summarized in a series of maps of the 50 states. Each map represents a single policy covariate, colored to reflect positive, negative, or no significant association. We expect differences across states, which would reinforce the current practice of decentralized comprehensive cancer control efforts, whereby each state has considerable autonomy to decide what goals and policies to pursue [38,39].

### Study Population

We define a cohort of persons aged 65+ in 2001 with both Parts A and B Medicare for the entire period 2001-2005, and we follow these individuals over time, using 100% Medicare claims annually to record any endoscopy use by persons. Persons included in the cohort must remain alive during the entire period and remain living in the same state. Table 2 provides sample statistics by state, where the number of cohort observations per state is reported. The smallest cohort is in Alaska (22,585 individuals) and the largest is in Florida (1,139,258).

**Table 2:** Cohort Size Used in Regression Models, and Number of PCSAs and Counties in Each of 50 States, and US Totals

State	Number of persons in population cohort	Number of PCSAs	Number of counties	State	Number of persons in population cohort	Number of PCSAs	Number of counties
Alabama	319,335	144	67	Montana	79,539	71	56
Alaska	22,585	24	27	Nebraska	146,001	121	93
Arizona	223,305	74	15	Nevada	75,709	30	17
Arkansas	224,275	149	75	New Hampshire	95,298	46	10
California	1,126,335	338	58	New Jersey	567,836	139	21
Colorado	156,466	96	63	New Mexico	100,328	61	33
Connecticut	245,186	71	8	New York	1,040,451	324	62
Delaware	64,072	12	3	North Carolina	587,505	207	100
Florida	1,139,258	167	67	North Dakota	62,867	71	53
Georgia	464,828	169	159	Ohio	783,948	254	88
Hawaii	56,573	23	5	Oklahoma	248,870	156	77
Idaho	82,703	57	44	Oregon	151,816	78	36
Illinois	818,437	258	102	Pennsylvania	819,431	296	67
Indiana	471,278	172	92	Rhode Island	50,326	14	5
Iowa	274,939	225	99	South Carolina	308,796	110	46
Kansas	211,602	162	105	South Dakota	72,116	95	66
Kentucky	307,484	145	120	Tennessee	395,590	145	95
Louisiana	244,130	112	64	Texas	1,118,495	414	254
Maine	121,387	91	16	Utah	113,066	54	29
Maryland	346,573	62	24	Vermont	50,631	49	14
Massachusetts	362,711	107	14	Virginia	492,814	170	128
Michigan	765,461	191	83	Washington	314,345	119	39
Minnesota	314,019	176	87	West Virginia	162,307	123	55
Mississippi	211,398	141	82	Wisconsin	412,030	173	72
Missouri	387,278	213	115	Wyoming	37,384	41	23
**Total US**	**17,249,117**	**6,740**	**3,133**				

Note: PCSA = Primary Care Service Area

### Statistical Analysis

The outcome of interest is endoscopic procedure utilization by our sample cohorts, identified from their Medicare claims. Any type or amount of endoscopic procedure use over the 5-year period was used to define a binary indicator to use as the outcome variable for each person. We estimate a fully adjusted multilevel probit regression model of individual screening behavior, which includes individual-level demographic variables; local neighborhood variables, including poverty, residential segregation, and English language ability among the elderly; and county-level health care system factors, such as MMC penetration, provider density, and distance to closest endoscopy provider (see Table 1 for variables included in modeling). Recognizing that individual state's comprehensive cancer control efforts, political, and regulatory environments are unique, we examine states separately. Because MMC plans may have used federal subsidies to enter riskier markets where elderly had lower coverage rates for Part B Medicare during this period [40,41], we expect that there may be a contemporaneous negative association between MMC penetration and area endoscopy utilization rates. Thus, we lag the managed care penetration variable 3 years (using 1998 data to predict 2001-2005 utilization) to reduce the potential for endogeneity caused by this sort of market selection by the MMC plans.

We used generalized estimating equations (GEE) to adjust the standard errors of area-level variables to reduce the bias caused by repeated measures for all people in an area. The GEE approach is appropriate when the outcome variable is binary and when researchers are concerned with estimating population-level effects (rather than community-specific effects) [42-44]. The estimated regression slope parameters, estimating the association between explanatory and outcome variable, are interpreted as marginal probability impacts. The estimate 0.05 on covariate X, for example, is interpreted as follows: a small increase in covariate × is associated with a 5% increase in the probability of endoscopic CRC screening utilization in the state population, on average, holding all other covariates constant statistically.

**Figure 3:**
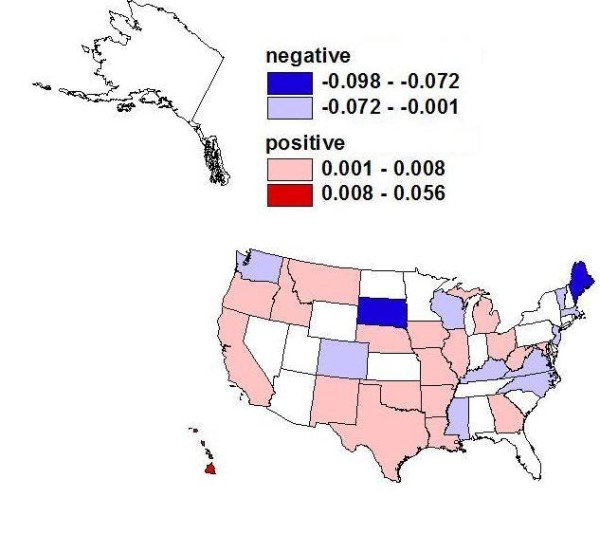
**Medicare Managed Care (MMC) Penetration and Probability of Endoscopic Colorectal Cancer Screening**. Findings from state-specific multivariate regressions: Associations between Medicare managed care (MMC) penetration and probability of endoscopic colorectal cancer screening in the 100% Medicare fee-for-service (FFS) population cohort defined in 2001 and followed through 2005.

**Figure 4:**
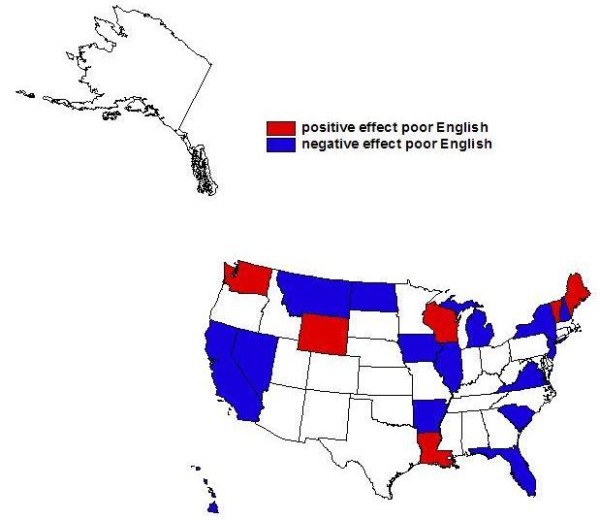
**English Language Ability and Probability of Endoscopic Colorectal Cancer Screening**. Findings from state-specific multivariate regressions: Associations between proportion elderly with poor english language ability in one's neighborhood and probability of endoscopic colorectal cancer screening in the 100% Medicare fee-for-service (FFS) population cohort defined in 2001 and followed through 2005.

**Figure 5:**
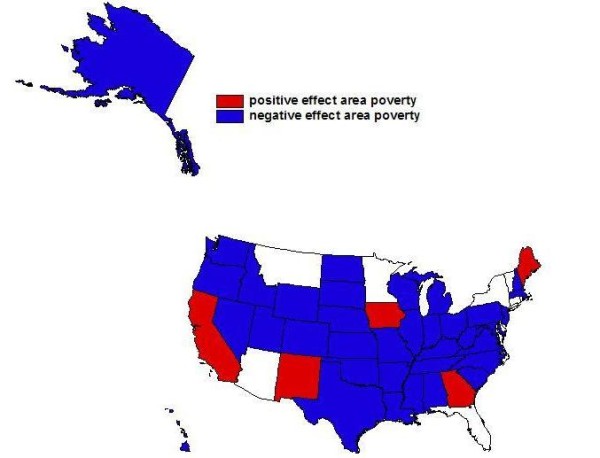
**Poverty and Probability of Endoscopic Colorectal Cancer Screening**. Findings from state-specific multivariate regressions: Associations between poverty in one's neighborhood and probability of endoscopic colorectal cancer screening in the 100% Medicare fee-for-service (FFS) population cohort defined in 2001 and followed through 2005.

Estimating a separate regression for each of the 50 states resulted in a large volume of empirical findings. We translate the particular findings of interest using three maps of the United States to depict the slope parameter estimates for the three covariates of central focus (Figures 3, 4, and 5). The maps display 50 slope parameter estimates for each covariate, because there is a separate estimate for each state. When the estimated slope parameter is not significantly different from zero, it is represented as zero/no effect. Using this innovative format for spatial translation of the research findings allows the large volume of findings to be condensed to manageable subsets that can be compared visually across the states.

## Discussion

We focus on the findings relevant to the three main research questions posed above and present these graphically using maps of the United States. In answer to the first research question, we find that there are significant MMC spillover effects and that these do vary across the states (see Figure 3). The findings presented here based on the 100% Medicare FFS population provide the first evidence to date for the entire Medicare population following benefits expansion in 2001 to cover colonoscopy. Prevalence of MMC in one's neighborhood was associated with lower probability of screening in 12 states (negative spillover on the FFS population) and higher probability in 19 states (positive spillover on the FFS population) (see Figure 3, Table 3). Consistent with our theory regarding managed care plan promotion of endoscopy dependent on ability to recoup financial benefits, we find that the state with the largest positive spillover effect (5.6% higher probability of screening in more penetrated markets) is Hawaii. Hawaii has had nearly universal employer-mandated health insurance since 1974. In Hawaii, 48% of the insured population was in managed care (health maintenance organization [HMO]) plans in 2008, ranking as the top state in terms of managed care penetration (see Table 3). In this state, managed care plans are more likely to be able to reap the benefits in terms of cost savings for cost-effective technologies because long-term enrollment in the plan is more likely under the employer-mandated system. Also, with few people uninsured, Hawaii's health care costs per person are lower than any other state's because the population receives timely preventive care services [45]. Thus, it is not surprising that the estimated MMC spillover effect is the largest and most positive for this state. Several other states with positive spillovers have also had historically high managed care penetration, indicating acceptance of the managed care model by their state populations, most notably California (see Table 3). However, not all states with positive spillovers show a long history of managed care acceptance.

**Table 3:** Managed Care (HMO) Penetration of Insured Population, by State, 1994-2008

State	1994	1998	2008	State	1994	1998	2008
Alaska	0.000	0.000	0.002	**Montana**	0.015	0.034	0.059
Alabama	0.100	0.107	0.041	*North Carolina*	0.095	0.166	0.053
**Arkansas**	0.038	0.111	0.030	North Dakota	0.083	0.029	0.026
Arizona	0.358	0.319	0.253	**Nebraska**	0.011	0.157	0.046
**California**	**0.383**	**0.454**	**0.429**	New Hampshire	0.170	0.350	0.113
*Colorado*	0.244	0.354	0.196	*New Jersey*	0.169	0.340	0.208
Connecticut	0.274	0.377	0.215	**New Mexico**	0.174	0.319	0.266
Delaware	0.205	0.344	0.192	Nevada	0.147	0.271	0.200
Florida	0.201	0.263	0.194	New York	0.243	0.363	0.277
**Georgia**	0.088	0.159	0.192	**Ohio**	0.192	0.225	0.179
**Hawaii**	**0.232**	**0.320**	**0.477**	**Oklahoma**	0.073	0.135	0.066
**Iowa**	0.041	0.099	0.075	**Oregon**	0.375	0.397	0.271
**Idaho**	0.012	0.082	0.046	Pennsylvania	0.215	0.351	0.280
**Illinois**	0.169	0.235	0.123	Rhode Island	0.288	0.360	0.202
Indiana	0.074	0.161	0.170	South Carolina	0.042	0.115	0.101
Kansas	0.109	0.150	0.147	*South Dakota*	0.029	0.049	0.100
*Kentucky*	0.121	0.177	0.080	Tennessee	0.162	0.238	0.255
**Louisiana**	0.070	0.162	0.075	**Texas**	0.097	0.177	0.132
*Massachusetts*	0.352	0.502	0.343	Utah	0.192	0.348	0.296
**Maryland**	0.362	0.360	0.261	*Virginia*	0.084	0.210	0.163
*Maine*	0.062	0.238	0.094	*Vermont*	0.126	0.289	0.088
**Michigan**	0.202	0.248	0.277	*Washington*	0.164	0.283	0.186
Minnesota	0.266	0.306	0.213	*Wisconsin*	0.242	0.297	0.248
**Missouri**	0.147	0.268	0.132	**West Virginia**	0.000	0.102	0.150
*Mississippi*	0.003	0.034	0.015	Wyoming	0.000	0.028	0.042

Note: **Bold font **indicates the 19 states with positive MMC spillovers shown in Figure 3.

*Italics font *indicates the 12 states with negative MMC spillovers shown in Figure 3.

Sources: (1994 and 1998 data) InterStudy, "The InterStudy Competitive Edge: HMO Report," St. Paul, Minnesota; (2008 data) Kaiser Family Foundation, State Health Facts, retrieved June 15, 2010, from http://www.statehealthfacts.org/comparemaptable.jsp?cat=7&ind=349

In answer to the second research question, we find that there are significant elderly language ability effects and that these vary across the states. These effects are positive in 6 states (Louisiana, Maine, Vermont, Washington, Wisconsin, and Wyoming) and negative in 15 states (Alaska, California, Florida, Hawaii, Illinois, Iowa, Michigan, Montana, Nevada, New Hampshire, New Jersey, New York, North Dakota, South Carolina, and Virginia) (see Figure 4). These findings have implications for intervention policies, which could target multilingual health communication efforts to areas with lower English proficiency among the elderly in the states where language ability effects are negative, to boost endoscopic CRC screening rates.

In answer to the third research question, we find that there are significant area poverty effects and that these vary across the states. Perhaps surprisingly, these effects are positive in 5 states (California, Georgia, Iowa, Maine, and New Mexico) (see Figure 5). They are not significantly different from zero in 9 states and are significantly negative in the remaining states. The positive poverty effect noted for Georgia may be explained by the fact that two Centers for Disease Control and Prevention (CDC)- funded intervention projects to promote CRC screening among low-income groups were implemented in Georgia during our research period [38]. More recently, CDC has launched the Colorectal Cancer Control Program (CRCCP), which provides funding to 22 states for 5 years. Under this funding program, local comprehensive cancer control programs provide CRC screening to low-income people aged 50 to 64 years when no other payment option is available. Although the CRCCP was not launched during our study period, the remaining 4 states with a positive poverty coefficient (California, Iowa, Maine, and New Mexico) are now participants in this new program [46]. This suggests that these states have been proactive in the comprehensive cancer control planning as regards outreach to poor people in their states.

## Conclusions

The socio-ecological conceptual model used as the basis for our empirical work (see Figure 1, Table 1) includes a comprehensive set of multilevel factors that might impact a person's utilization of CRC screening. From a statistical perspective, it is important to include a comprehensive set of covariates so that the independent effects of several contextual factors of interest can be assessed while holding other factors constant. We focus on three community and health system factors here, with particular focus on spillover effects from MMC penetration on the FFS-insured individuals we study.

In a recent paper using 1999 data, Koroukian et al. [23] analyzed CRC screening spillovers from MMC on the FFS population in the 2,655 largest US counties. They found consistently and significantly positive MMC penetration spillovers on utilization of sigmoidoscopy alone and colonoscopy following FOBT or sigmoidoscopy, but no consistent findings for colonoscopy alone, the largest utilization group, which is not surprising because Medicare benefits had not yet been expanded to cover colonoscopy in 1999. Pooling all counties together, they concluded that there could be modest MMC spillovers that improve use of CRC screening by FFS enrollees. Understanding and disentangling the mechanisms that impact MMC spillovers is a complex endeavor, and prior studies have indicated that there are numerous interacting factors [47]. For instance, patient self-selection is an essential aspect to consider when comparing patterns of preventive care usage between the FFS and MMC populations. Therefore, differences in the patient population enrolled in a health plan may impact the patterns and selection of screening tests [17,48].

Our paper is the first to use 100% Medicare population data from all counties in 50 states to provide a definitive picture of MMC spillovers on FFS Medicare insured individuals following expansion of benefits to cover colonoscopy in 2001. We find that MMC spillover effects are positive in more states than they are negative (19 versus 12, respectively). Our findings may differ from Koroukian et al. [23] for several reasons: differences in time period studied (1999 vs. 2001-2005), differences in conceptual modeling, differences in the geographic coverage of the studies, differences in the treatment of spatial heterogeneity, and differences in stability of the MMC market. In particular, Koroukian et al. [23] examined only a subset of the largest US counties, pooled together, whereas we include all counties and perform a truly 100% population analysis, examining each state separately to reflect the heterogeneity inherent in decentralized state-run comprehensive cancer control efforts.

We find that the largest positive MMC spillover occurs in Hawaii. Hawaii is unusual in that it mandated universal employer-provided insurance decades ago and has very low uninsured population rates and the highest HMO penetration rates in the country. Hawaii also enjoys the lowest health care costs per capita, and our findings suggest that managed care plans in this environment are able to reap the financial rewards from promoting cost-increasing endoscopy technology that reduces morbidity and mortality burdens.

Our evidence suggests negative MMC spillovers in 12 states (Colorado, Kentucky, Maine, Massachusetts, Mississippi, New Jersey, North Carolina, South Dakota, Vermont, Virginia, Washington, and Wisconsin). Negative spillovers are important for health policy because they reflect detrimental health behavioral effects for persons not insured by managed care, who live in areas with greater managed care presence. Because CRC screening is already lower than optimal, these findings are of considerable policy importance.

Our MMC spillover findings may be an artifact of the time period studied. More research on MMC spillovers is needed because this period was one of considerable turmoil in MMC markets. Following the Balanced Budget Act of 1997, many MMC plans withdrew from certain counties where profit margins were thin, leaving former plan members involuntarily disenrolled [40]. Then, in 2003, the Medicare Modernization Act promoted new demonstration plans in some states with limited drug coverage to re-stimulate interest in MMC plans among the elderly, which had limited success [41]. Future analysis from 2006 forward will be enlightening, as the Part D drug benefit was implemented and all MMC plans (now called Medicare Advantage plans) were required to offer drug coverage. This coverage expansion caused revived interest in Medicare managed care plans among the elderly. Drug coverage may relax the out-of-pocket constraints that face financially insecure elderly and promote use of endoscopic procedures. Time will tell whether the Medicare managed care spillover effects remain variable over geography, or whether our findings were a symptom of upheaval in the MMC plan market.

We attempted to include relevant variables at the individual, neighborhood, and health system levels in the multivariate regressions. Although we do have information regarding eligibility for state assistance paying Part B premiums (which is available for low-income seniors), not all low-income seniors apply for this. Socioeconomic status at the individual Medicare beneficiary level was not available, and this is a potential limitation of the analysis. Also, colonoscopy is recommended every 10 years for older persons of average risk [19]. However, our study examines the screening behavior of the Medicare population for 5 years (2001-2005) following the expansion of benefits to cover colonoscopy. It is likely that some of the population being studied received endoscopic screening prior to 2001 or after 2005. Because we examine a 5-year time window, our findings may underestimate the proportion of persons utilizing endoscopy commensurate with established guidelines. In addition, the Medicare Modernization Act of 2003 could have introduced changes in MMC screening practices and their spillovers midway through the study period. Finally, we lagged the MMC penetration variable by 3 years to avoid potential endogeneity caused by plan selection of certain types of markets for entry, and this may have introduced measurement error, as MMC penetration rates were declining in many counties during the study period.

Despite these limitations, this paper provides important evidence to inform the ongoing debate regarding health care market reform in the United States. Our findings demonstrate that access to and utilization of endoscopic CRC screening, even among a well-insured elderly population, depends on community-level social determinants (such as English language ability among the elderly) in addition to market factors (area poverty, which drives both supply and demand for endoscopy; and spillover effects from managed care). Of policy importance, contextual variables like these can be used to target communities in greatest need of health care interventions to reduce information problems or potential cultural or financial barriers in order to improve utilization of technology that can both detect and prevent CRC. Thus, the importance of space and place is not only of scientific interest, but is also central to the effective design of policies, as reflected in the August 11, 2009, White House Memorandum on "Developing Effective Place-Based Policies for the FY-2011 Budget" [49]. This study confirms recent findings regarding barriers and facilitators to endoscopy utilization by older persons, such as having dual coverage of Medicare and Medicaid, being female, being African American or Hispanic [50], and living in rural versus urban areas [51]. This is the first study to date to examine the socio-ecological context of endoscopy utilization in the entire Medicare FFS population, providing information that is actionable for interventions that are as heterogeneous as their state environments.

## Abbreviations

CDC: Centers for Disease Control and Prevention; CRC: colorectal cancer; CRCCP: colorectal cancer control program; FFS: fee-for-service; FIT: fecal immunochemical test; FOBT: fecal occult blood test; GEE: generalized estimating equations; HMO: health maintenance organization (managed care plan); MMC: Medicare managed care; PCSA: primary care service area; US: United States.

## Competing interests

The authors declare that they have no competing interests.

## Authors' contributions

LM, MK, and MU have made substantial contributions to the acquisition, analysis, and interpretation of data. LM and SS have been involved in drafting the manuscript or revising it critically for important intellectual content. All authors have given final approval of the version submitted for consideration for publication by the *International Journal of Health Geographics*.
